# Climate change-induced vegetation change as a driver of increased subarctic biogenic volatile organic compound emissions

**DOI:** 10.1111/gcb.12953

**Published:** 2015-05-21

**Authors:** Hanna Valolahti, Minna Kivimäenpää, Patrick Faubert, Anders Michelsen, Riikka Rinnan

**Affiliations:** 1 Terrestrial Ecology Section, Department of Biology, University of CopenhagenCopenhagen, Denmark; 2 Center for Permafrost (CENPERM), Department of Geography and Geology, University of CopenhagenCopenhagen, Denmark; 3 Department of Environmental Sciences, University of Eastern FinlandKuopio, Finland; 4 Chaire en éco-conseil, Département des sciences fondamentales, Université du Québec à ChicoutimiChicoutimi, QC, Canada

**Keywords:** Arctic, BVOCs, climate change, isoprene, monoterpene, plant volatiles, sesquiterpene, temperature, vegetation change

## Abstract

Emissions of biogenic volatile organic compounds (BVOCs) have been earlier shown to be highly temperature sensitive in subarctic ecosystems. As these ecosystems experience rapidly advancing pronounced climate warming, we aimed to investigate how warming affects the BVOC emissions in the long term (up to 13 treatment years). We also aimed to assess whether the increased litterfall resulting from the vegetation changes in the warming subarctic would affect the emissions. The study was conducted in a field experiment with factorial open-top chamber warming and annual litter addition treatments on subarctic heath in Abisko, northern Sweden. After 11 and 13 treatment years, BVOCs were sampled from plant communities in the experimental plots using a push–pull enclosure technique and collection into adsorbent cartridges during the growing season and analyzed with gas chromatography–mass spectrometry. Plant species coverage in the plots was analyzed by the point intercept method. Warming by 2 °C caused a 2-fold increase in monoterpene and 5-fold increase in sesquiterpene emissions, averaged over all measurements. When the momentary effect of temperature was diminished by standardization of emissions to a fixed temperature, warming still had a significant effect suggesting that emissions were also indirectly increased. This indirect increase appeared to result from increased plant coverage and changes in vegetation composition. The litter addition treatment also caused significant increases in the emission rates of some BVOC groups, especially when combined with warming. The combined treatment had both the largest vegetation changes and the highest BVOC emissions. The increased emissions under litter addition were probably a result of a changed vegetation composition due to alleviated nutrient limitation and stimulated microbial production of BVOCs. We suggest that the changes in the subarctic vegetation composition induced by climate warming will be the major factor indirectly affecting the BVOC emission potentials and composition.

## Introduction

Emissions of biogenic volatile organic compounds (BVOCs) from subarctic ecosystems have been observed to be highly responsive to temperature (Tiiva *et al*., 2008; Faubert *et al*., 2010; Holst *et al*., 2010; Potosnak *et al*., 2013; Rinnan *et al*., 2014). In order to be able to better predict the consequences of climate change on the subarctic BVOC emissions, we need to know whether the plant communities and their emission rates acclimate to the increasing temperature in the long term.

BVOCs are a diverse group of compounds released from the vegetation and soil. Their lifetime in the atmosphere varies from minutes to several days (Kesselmeier & Staudt, 1999). The most widely studied single BVOC is isoprene (C_5_H_8_), which together with monoterpenes (C_10_H_16_) and sesquiterpenes (C_15_H_24_) belongs to the biochemical class of terpenoids. The annual global BVOC emission rate is estimated to be 700–1000 × 10^12^ g C (Laothawornkitkul *et al*., 2009), of which isoprene accounts for 440–660 × 10^12^ g C (Guenther *et al*., 2006). The modeled emissions of isoprene and monoterpenes from the Arctic, defined as the area north of 60°N, contribute to one and two percent of the total annual emissions globally, respectively (Sindelarova *et al*., 2014). The emission of isoprene and some monoterpenes is mainly dependent on light intensity, and the emission of all terpenoids is strongly dependent on temperature (Kesselmeier & Staudt, 1999). BVOCs are important in defense against abiotic and biotic stresses and in plant-to-plant and plant-to-insect communication (Frost *et al*., 2008).

BVOCs are very reactive compounds and they play a significant role in atmospheric chemistry. Photo-oxidation of BVOCs forms condensable compounds, which further leads to the formation of secondary organic aerosols (SOA) (Claeys *et al*., 2004). SOA particles are able to act as cloud condensation nuclei, potentially increasing the reflectivity of clouds, and it has therefore been suggested that they exert a negative radiative effect (i.e., a cooling) on the climate (Spracklen, 2008; Pöschl *et al*., 2010; Scott *et al*., 2014). BVOCs interact with anthropogenic NO_x_ under sunlight, forming tropospheric ozone (O_3_) (Atkinson, 2000). In Arctic and subarctic regions where NO_x_ levels are relatively low, BVOCs mainly affect the atmospheric chemistry by reacting with the OH radicals, which can increase the lifetime of methane, an important greenhouse gas (Di Carlo *et al*., 2004).

The annual temperature in the area north of 60°N is projected to increase by 4–9 °C by year 2100, depending on the assumed socioeconomic scenario, which is more than twice as much as the global mean temperature increase (IPCC, 2013). Mean annual temperature in the northern high latitudes has already increased by 2–3 °C since the 1950s (ACIA, 2005). These warmer conditions together with a prolonged growing season will bring significant changes to ecosystem dynamics in the boreal and subarctic regions (Peñuelas & Filella, 2001).

Global warming generates a pressure for arctic and subarctic plant species to adapt to the new conditions. The ongoing warming has, for example, increased plant growth in recent decades (Myneni *et al*., 1997) and led to expansion of shrubs (Chapin III *et al*., 2005) and treeline of mountain birch (*Betula pubescens* ssp. *czerepanovii*) to higher altitudes (Truong *et al*., 2007). The expansion of vegetation, especially shrubs and trees, decreases the albedo and is predicted to generate new feedback loops, for example, by amplifying atmospheric warming in the future (Chapin *et al*., 2005). Increased plant growth and the increasing abundance of deciduous plant species increase the leaf litter fall (Cornelissen *et al.,* 2007). This greater amount of leaf litter increases the microbial activity in the soil and the amount of available nutrients for plants (Rinnan *et al*., 2008). Warming has been shown to favor deciduous and evergreen shrubs, graminoids and forbs by increasing either their abundance or their maximum height and at the same time disfavor the abundance of bryophytes (Graglia *et al*., 2001; Elmendorf *et al*., 2012a) due to shading by the more abundant vascular plants (Cornelissen *et al*., 2001; Lett & Michelsen, 2014). Different plant species emit unique bouquets of BVOCs (Kesselmeier & Staudt, 1999), and therefore changes in vegetation composition can impact on the community-level BVOC emissions.

An *in situ* manipulation experiment using passive warming by open-top greenhouses setup on a subarctic heath showed that a mere 2 °C warming doubled the emissions of mono- and sesquiterpenes and increased isoprene emission by 50–80% after 7–8 years of exposure (Tiiva *et al*., 2008; Faubert *et al*., 2010). These studies concluded that the increase in BVOC emissions was due to a direct warming effect rather than vegetation changes. The aim of this study was to assess whether warming for an additional 5 years has led to an acclimation of the vegetation to the changed abiotic conditions in the same experimental site and, consequently, less pronounced treatment effects on BVOC emission, or to a change in the vegetation composition and marked changes in BVOC release. The experiment also permits analysis of the effects of enhanced leaf litter supply. The previous studies concluded that an annual addition of mountain birch (*B. pubescens* ssp. *czerepanovii*) litter had no or minor effects on the emission of volatiles (Tiiva *et al*., 2008; Faubert *et al*., 2010). Here, we will evaluate whether the litter addition treatment has longer-term effects on the BVOC emissions from the ecosystem – either directly or via effects on the vegetation composition.

## Materials and methods

### Study site and experimental design

**Figure 1:**
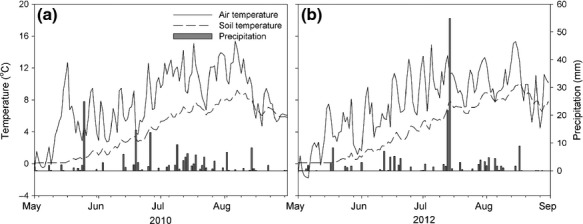
Daily precipitation, soil and air temperature for the growing seasons 2010 (a) and 2012 (b). Temperature and precipitation data were collected every hour and provided by Abisko Scientific Research Station.

The experimental site was a wet subarctic heath located in Abisko, northern Sweden (68°21′N, 18°49′E, 385 m a.s.l). Mean annual temperature and precipitation (2002–2011) are 0 °C and 332 mm, respectively (Callaghan *et al*., 2013). Temperature and precipitation data for the years under investigation here are presented in Fig.1. Vegetation at the experimental site is dominated by evergreen and deciduous dwarf shrubs, graminoids and forbs. The highly organic soil is covered by *Sphagnum warnstorfii*, other moss species and lichens (Lett & Michelsen, 2014). Soil characteristics have been reported by Rinnan *et al*. (2008).

The experiment mimicking climatic warming and increasing litter fall following from the ongoing changes in tundra vegetation (Chapin *et al*., 2005; Truong *et al*., 2007; Cornelissen *et al*., 2007) has been maintained in the area since 1999. This experiment consisted of control (*C*), warming (*W*), litter addition (*L*) and a combination of warming and litter addition (*W* + *L*) treatments (Rinnan *et al*., 2008). Each treatment was replicated in six blocks yielding 24 plots of 120 × 120 cm within an area of 1000 m^2^.

The warming treatment consisted of open-top tents made of transparent plastic, which increase the air temperature by 2 °C and the surface soil temperature by 1–2 °C (Rinnan *et al*., 2008; unpublished data from 2009). They also reduce photosynthetically active radiation (PAR) by 10%. During the current measurements, the warming treatment increased the chamber temperature on average by 0.5 °C (Table S1). The plastic tents were erected every year in late May–early June and removed after the field season in late August–early September. In the litter addition treatment, 90 g DW m^−2^ of air-dried mountain birch (*Betula pubescens* ssp. *czerepanovii* (N.I. Orlova) Hämet-Ahti) litter was added every autumn. This corresponds with quality and amount of the annual litter fall in a nearby forest, dominated by *B. pubescens* ssp. *czerepanovii*, and simulates thus well the expected increase in deciduous species (Bylund & Nordell, 2001; Cornelissen *et al*., 2007).

### Vegetation analysis

Species coverage and composition in a 220 × 220 mm area in each plot were analyzed in early August by the point intercept method (Jonasson, 1988). Briefly, a pin was passed through 100 holes in a transparent polycarbonate plate and the species was recorded each time it was touched by the pin. Plant species were grouped as graminoids, deciduous shrubs, evergreen shrubs, forbs, vascular cryptogams, mosses and lichens.

### Sampling of BVOCs

BVOC emissions were sampled 8 times in 2010 and 4 times in 2012 during the growing season in the same area as used for vegetation analysis. Samplings were made using transparent polycarbonate chambers (thickness 1.5 mm, 220 × 220 mm, height 200 mm; Vink Finland, Kerava, Finland) placed on an aluminum collar permanently installed in each plot in 1999. Collar grooves were filled with water before placing the chamber to create an airtight headspace inside the chamber. Before the 30-min-long sampling, the chamber was flushed for 10 min with a flow rate of 1000 ml min^−1^ to replace the headspace with filtered air (Ortega & Helmig, 2008). During the sampling, the air was circulated through the chambers using battery-operated pumps (12V; Rietschle Thomas, Puchheim, Germany) at 200 ml min^−1^ for both inflow and outflow and the chambers were equipped with fans to ensure well-mixed headspace. Incoming air was purified using a charcoal filter (Wilkerson F03-C2-100, Mexico) to remove particles and volatile impurities and a MnO_2_ scrubber (Ozone Scrubber Cartridge, Environnement S.A. France) to remove ozone (Fig. S1).

The BVOCs released from the plots were trapped in stainless steel adsorbent tubes (150 mg Tenax TA, 200 mg Carbograph 1TD, Markes International Limited, Llantrisant, UK). After the collection, the tubes were sealed with Teflon-coated brass caps and stored at 5 °C until analysis.

Temperature and relative humidity inside the chamber were recorded (Hygrochron DS 1923-F5 iButton, Maxim Integrated Products Inc., CA, USA) once a minute during the sampling. PAR was recorded every 10 s using PAR sensors (S-LIA-M003, Onset Computer Corporation, Bourne, MA, USA) coupled to a Hobo Micro Station (Onset Computer Corporation); see Table S1 for chamber temperature and PAR during measurements.

### BVOC analysis

The BVOCs collected in adsorbent tubes were analyzed using gas chromatography–mass spectrometry (GC-MS) following thermal desorption. In 2010, the analysis was carried out on a Hewlett Packard instrument (GC type 6890, MSD 5973, Palo Alto, CA, USA). After thermodesorption at 250 °C for 10 min and cryofocusing at −30 °C with an automated thermal desorber (Perkin Elmer ATD400, Wellesley, MA, USA), the samples were immediately injected into an HP-5 capillary column for separation (length 50 m × ø0.2 mm × 0.33 *μ*m film thickness). The temperature was held at 40 °C for 1 min; then raised to 210 °C at a rate of 5 °C min^−1^. Helium was used as a carrier gas. In 2012, the samples were analyzed using Unity 2 thermal desorber coupled with an Ultra autosampler and an Agilent GC-MS (7890A Series GC, 5975C inert MSD/DS Performance Turbo EI, Agilent Technologies, Santa Clara, CA, USA). The column and the method used were identical to the year 2010 except for the cryofocusing temperature, which was −10 °C.

BVOCs were identified using pure standards and according to their mass spectra in the NIST data library and quantified by pure standard solutions for isoprene, *α*-pinene, camphene, sabinene, 3-carene, limonene, eucalyptol, *γ*-terpinene, copaene, *δ*-cadinene, aromadendrene, 2-methylfuran and cis-3-hexenyl acetate (Fluka, Buchs, Switzerland) based on total ion counts (TIC). Detection limit was approximately 1 ng. When quantifying compounds for which no pure standard was available, *α*-pinene was used for quantification of monoterpenes, copaene for sesquiterpenes and cis-3-hexenyl acetate for other volatile organic compounds. Chromatograms were analyzed using the software enhanced chemstation (Agilent Technologies). Compounds which had an identification quality above 90% with the NIST data library and which were present in at least 10% of the samples were accepted in the dataset.

BVOC emission rates were expressed on ground area basis (*μ*g m^−2^ h^−1^) (see Faubert *et al*., 2012 for description of calculations), where the surface topography of each plot was taken into account when determining the chamber volume. The emission potentials of the terpenoids were calculated by standardizing to the temperature of 30 °C and the PAR of 1000 *μ*mol m^−2^ s^−1^ according to Guenther *et al*. (1993, 1995) to minimize the effects caused by differences in environmental conditions during sampling.

### Statistical analyses

We used mixed-models analysis of variance (anova) (ibm spss Statistics 19.0.0, SPSS Inc. IBM Company ©, Armonk, NY, USA) for testing the effects of warming, litter addition, date and their interactions on emissions of isoprene, total monoterpenes (MTs), total sesquiterpenes (SQTs) and total other VOCs. The block was included in the model as a random factor. The final model was obtained by excluding nonsignificant (cutoff level 0.2) effects, one by one, starting from the highest level interactions and highest probability values. Shapiro–Wilk’s normality test was used to check the normality of the data and the model residuals. In case the residuals were not normally distributed or data showed inhomogeneous variances, the data were logarithmic or square-root-transformed. Statistical analysis was run for both the actual emission data and emission potentials.

To test for the effects of warming and litter addition (including their interaction) on individual sampling dates, mixed-models anova was run for each sampling date. The sample plot was used as a unit of replication (*n *=* *6).

To test for treatment effects on the vegetation data, which did not fulfill the prerequisites of anova, we used the Kruskall–Wallis test followed by Mann–Whitney test for pairwise comparison with Bonferroni correction (i.e., *P*-values of Mann–Whitney tests multiplied by the number of tests). In all analyses, *P-*values < 0.05 were considered as statistically significant and those <0.1 to indicate a close to significant tendency.

Covariance between the emissions of individual BVOCs (dependent variables, *Y*) and plant species abundances (independent variables, *X*) was assessed with a partial least squares (PLS) regression analysis using SIMCA 13.0.3 (Umetrics, Umeå, Sweden). The PLS analysis was performed for the BVOC measurement date closest to the plant analysis date for both 2010 and 2012 together. Cross-validation was done according to the block.

## Results

### BVOC emissions

**Figure 2:**
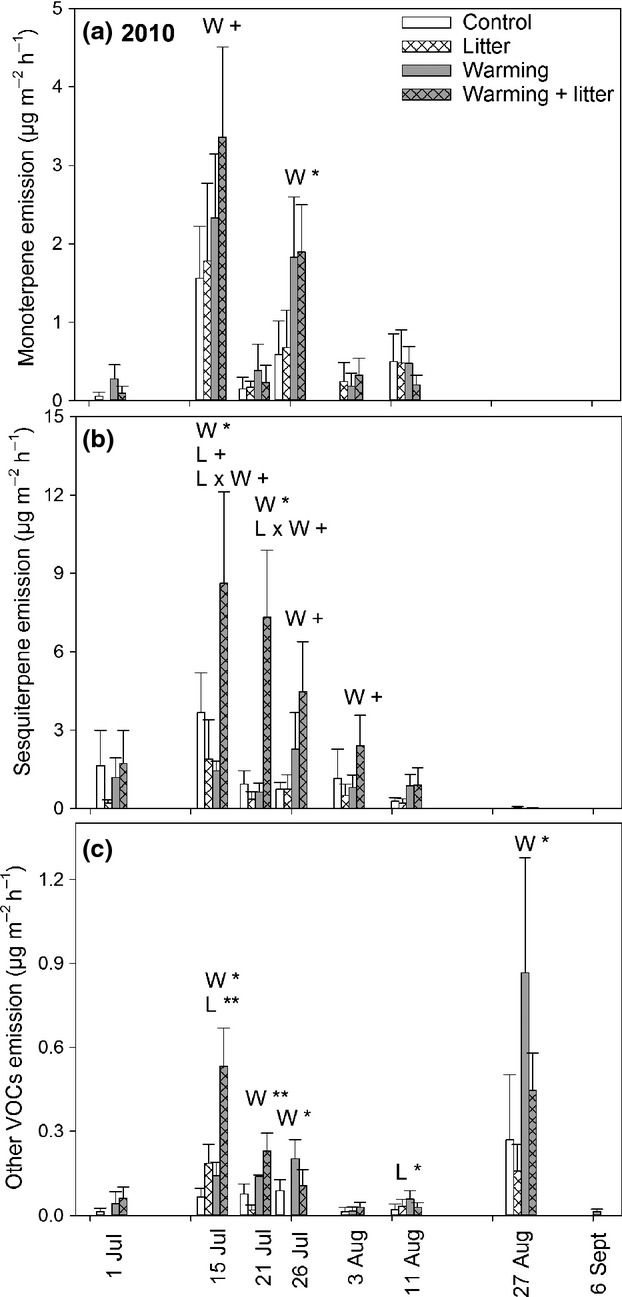
Biogenic volatile organic compound (BVOC) emissions from a subarctic tundra heath in 2010. Figure presents nonstandardized emissions of monoterpenes (a), sesquiterpenes (b) and other VOCs (c) (mean ± SE; *n *=* *6) from control, litter addition, warming and combined treatments. Significant main effects of warming (*W*), litter addition (*L*) and their interaction (*L* × *W*) for mixed-models anovas are indicated by +*P *<* *0.1, *P <* *0.05 and **P <* *0.01 within a date. Note different *y*-axis scales.

**Figure 3:**
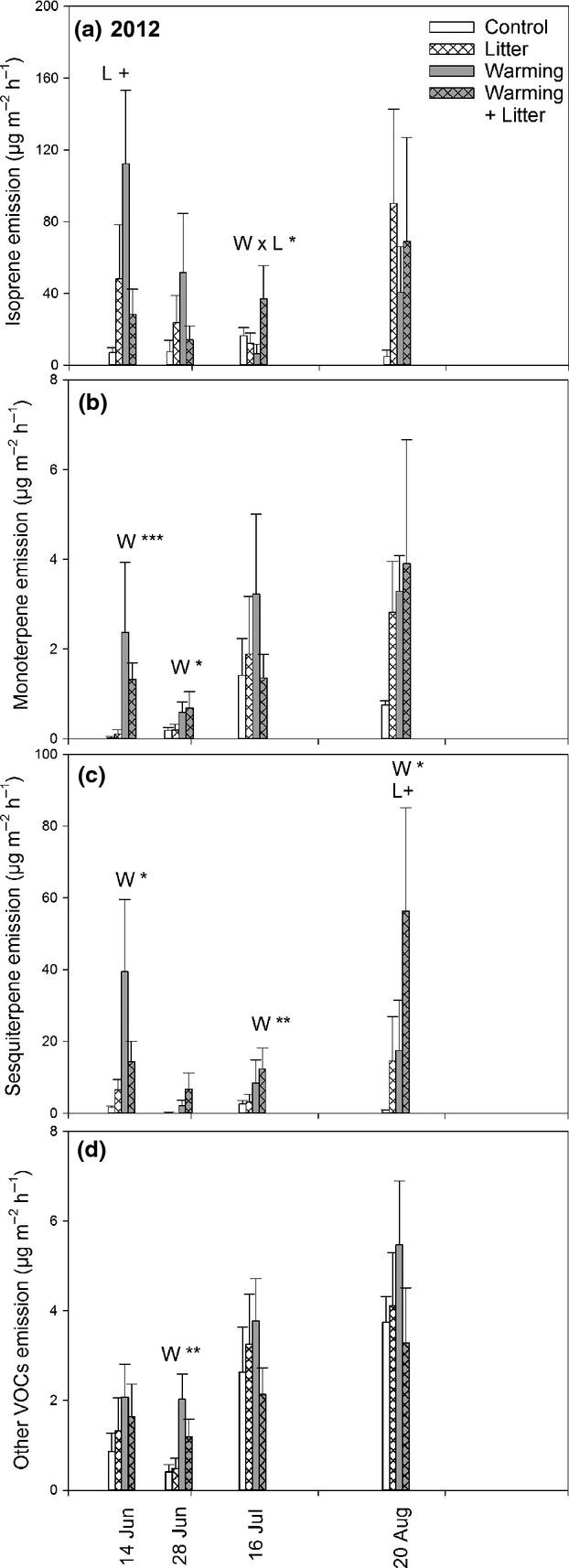
Biogenic volatile organic compound (BVOC) emissions from a subarctic tundra heath in 2012. Figure presents nonstandardized emissions of isoprene (a), monoterpenes (b), sesquiterpenes (c) and other VOCs (d) (mean ± SE; *n *=* *6) from control, litter addition, warming and combined treatments. Significant main effects of warming (*W*), litter addition (*L*) and their interaction (*L* × *W*) for mixed-models anovas are indicated by +*P *<* *0.1, *P <* *0.05, **P <* *0.01 and ***P <* *0.001 within a date. Note different *Y*-axis scales.

A total of 21 BVOCs (5 MTs, 12 SQTs and 4 other VOCs) were detected in 2010 and 30 compounds (7MTs, 15 SQTs and 8 other VOCs) in 2012, in addition to isoprene (see Table S2 for individual compounds). In both years, eucalyptol was the most emitted MT and *β*-selinene the most emitted SQT. The MTs 3-thujene and limonene as well as SQTs *α*-selinene and *α*-caryophyllene were also emitted in significant amounts. In 2010, hexane was the most emitted other VOC detected, and in 2012, toluene had the highest emission among other VOCs. The total MT emission rates in 2010 and 2012 were at a similar level, but the total SQT emissions were 3.5 times higher in 2012 than in 2010 (Figs2a,b and 3b,c). The most emitted single compound in 2012 was isoprene, which was left out of the 2010 results because of technical problems that prevented the analysis of <C6 compounds. The emission potentials for MT and SQT emissions (in 2012 also isoprene) are shown in Supplementary Table S3.

There was a three-way interaction effect on isoprene emission in 2012 showing that the effect of warming was dependent on the effect of litter addition and also on date (Table1; Fig.3a). This interaction was statistically significant in both actual and the emission potential data. In 2012, isoprene emission was increased by litter addition on June 14 and August 20 (Fig.3a). On July 16, the individual treatments decreased while the combined treatment increased the emission.

**Table 1:** anova table showing main effects and interactions of date (*D*), warming (*W*) and litter addition (*L*) on the emissions of isoprene (*I*), total monoterpenes (MT), total sesquiterpenes (SQT) and total of other VOCs in the actual (A) emissions and emission potentials (S). *P*-values for the main and interaction effects included in the model are shown

	*D*	*W*	*L*	*W* × *L*	*L* × *D*	*W* × *D*	*W* × *L* × *D*
2010							
MT_**A**_	<0.001	0.011	0.601	–	–	0.061	–
MT_**S**_	<0.001	0.007	0.864	–	–	–	–
SQT_**A**_	<0.001	<0.001	0.158	0.001	–	–	–
SQT_**S**_	<0.001	<0.001	0.279	0.005	–	–	–
Other VOCs	<0.001	<0.001	0.849	–	0.002	0.036	–
2012							
I_**A**_	0.323	0.323	0.469	0.656	0.054	0.572	0.032
I_**S**_	0.902	0.216	0.212	0.605	0.054	0.507	0.012
MT_**A**_	<0.001	<0.001	0.895	–	–	0.020	–
MT_**S**_	<0.001	<0.001	0.947	–	–	0.033	–
SQT_**A**_	0.002	<0.001	0.092	–	–	–	–
SQT_**S**_	0.065	<0.001	0.104	0.045	–	–	–
Other VOCs	<0.001	0.027	0.146	0.055	–	–	–

–, Factor not included in the model.

In both years, warming significantly increased the MT emissions and emission potentials (Table1, Figs2a, 3b and Table S4). In 2010, the increase was significant on July 15 and 26 (Fig.2a), and in 2012, on June 14 and 28 (Fig.3b). Litter addition alone or together with warming did not significantly affect the MT emissions rates.

SQT emission rates and emission potentials were also significantly increased by warming in both years (Table1, Figs2b and 3c). In 2010, the increase remained statistically significant or nearly significant from July 15 until August 3 (Fig.2b). In 2012, the warming effect was significant on June 14 and July 16, and August 20 (Fig.3c). While litter addition alone had no significant effects on SQT emissions in 2010, there was a significant *W* × *L* interaction over the season (Table1). In single campaigns, a marginally significant *W* × *L* interaction was found on July 15 and 21, because litter addition alone tended to decrease and the *W* + *L* treatment tended to increase the SQT emissions (Fig.2b). In 2012, litter addition tended to increase the SQT emissions (Table1), and this effect was most pronounced on August 20 (Fig.3c). The emission of other VOCs was also increased by warming in both years (Table1, Figs2c and 3d). In 2010, warming consistently increased the emissions on all dates except for July 1, August 3 and September 6, leading to a *W* × date interaction (Table1, Fig.2c). In 2012, the increase was significant on June 28 (Fig.3d). The effect of litter addition was not consistent. In 2010, litter addition significantly increased the emission of other VOCs on July 15 and August 11 with no clear effect on other dates (Fig.2c). In 2012, there was a marginally significant *W* × *L* interaction (Table1) because of the increase caused by warming alone, but less in combination with litter addition (Fig.3d).

**Figure 4:**
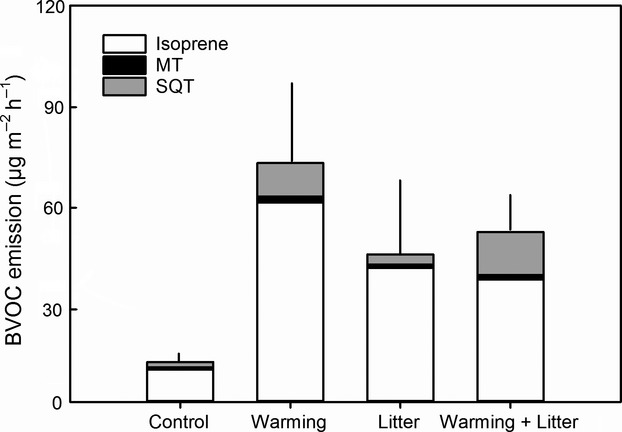
Contribution of isoprene, monoterpenes (MT) and sesquiterpenes (SQT) to total terpenoid emission averaged over all the measurements in 2010 and 2012. Note that isoprene data are only from 2012. Error bars indicate the standard error of mean for total emissions (*n *=* *6). See text for statistics.

When all the measurements for both 2010 and 2012 were averaged, the total terpenoid emissions were 2-fold in the warmed plots compared to the nonwarmed plots (Fig.4). The most drastic increase was observed for the SQT emissions, which increased 5-fold under warming (*P *=* *0.006). Warming increased the isoprene emission (2010 average) by 1.9 times (*P *=* *0.003) and MT emissions by 2 times (*P *=* *0.019).

### Vegetation coverage

In general, the most dominant vegetation group in all plots was the evergreen shrubs, followed by graminoids and deciduous shrubs (Tables2 and S5). The most dominant evergreen shrubs were *Empetrum hermaphroditum* and *Andromeda polifolia*. The graminoids present in the plots were mainly *Carex vaginata* and *Festuca ovina* while the deciduous shrubs were dominated by *Vaccinium uliginosum,* followed by *Betula nana*. In both years, the two most common forbs were *Astragalus alpinus* and *Tofieldia pusilla*. Two vascular cryptogam species, *Equisetum scirpoides* and *Equisetum arvense,* were found.

**Table 2:** Vegetation coverage (%, mean *± *SE, *n = *6) in control (*C*), litter addition (*L*), warming (*W*) and warming + litter addition (*W* + *L*) treatments in August 2010 and 2012

	*C*	*L*	*W*	*W* + *L*	*P*-value*
2010					
Graminoids	27.3 ± 2.3^a^	13.5 ± 2.4^b^	27.2 ± 2.6^a^	20.9 ± 1.7^a^	*<*0.001
Deciduous shrubs	18.1 ± 1.9^a^	18.7 ± 2.4^a^	28.7 ± 3.7^ac^	28.4 ± 2.7^bc^	0.006
Evergreen shrubs	45.0 ± 3.2^a^	50.8 ± 3.9^a^	49.1 ± 2.6^a^	63.2 ± 4.6^b^	0.034
Forbs	5.2 ± 0.5	9.7 ± 2.2	13.7 ± 4.4	4.8 ± 0.6	
Vascular cryptogams	2.3 ± 0.4	3.3 ± 0.7	2.8 ± 0.6	4.8 ± 1.1	
Total vascular plants	97.9 ± 2.6^a^	96.0 ± 5.0^b^	121.4 ± 5.6^a^	122.0 ± 7.7^b^	0.003
Moss	18.0 ± 3.3	27.1 ± 4.2	26.5 ± 3.7	27.9 ± 4.7	
Lichen	9.2 ± 1.3^a^	4.9 ± 1.6^b^	6.6 ± 1.2^a^	1.0 ± 0.3^c^	0.001
Litter	11.4 ± 1.7	13.1 ± 2.1	10.1 ± 1.6	16.6 ± 2.5	
2012					
Graminoids	23.6 ± 2.5^a^	11.0 ± 1.9^b^	24.7 ± 3.0^a^	20.8 ± 1.9^ab^	0.001
Deciduous shrubs	26.2 ± 2.2	22.3 ± 1.7	32.7 ± 6.4	23.4 ± 3.1	
Evergreen shrubs	47.0 ± 4.7	61.2 ± 6.5	57.2 ± 4.3	53.7 ± 4.2	
Forbs	5.4 ± 0.6^ac^	8.5 ± 1.4^ac^	14.5 ± 3.9^b^	3.9 ± 0.4^c^	0.020
Vascular cryptogams	4.6 ± 1.2	6.4 ± 1.3	6.3 ± 1.5	7.0 ± 1.6	
Total vascular plants	106.8 ± 6.1^a,b^	109.4 ± 6.7^a^	135.3 ± 9.4^b^	108.9 ± 8.2^a,b^	0.010
Moss	8.3 ± 2.1^a^	11.9 ± 1.9^a^	10.3 ± 2.0^a^	15.7 ± 3.0^b^	0.043
Lichen	7.5 ± 2.1^a^	5.3 ± 2.2^a^	3.5 ± 1.2^a^	0.2 ± 0.1^b^	0.001
Litter	15.5 ± 0.9	16.6 ± 2.1	16.0 ± 1.6	16.2 ± 1.6	

Values sharing a superscript letter within a plant group do not significantly differ from each other (*P *<* *0.05, Mann–Whitney test with Bonferroni correction).

* Statistically significant treatment effects by Kruskall–Wallis test.

The coverage of graminoids decreased in litter addition treatment in comparison with the other treatments (Table2). In 2010, the graminoid coverage in control plots was 2-fold compared to litter addition, and in 2012, the coverage was 2.5-fold. Litter addition decreased especially the coverage of *C. vaginata* in both years (Table S3).

In 2010, the coverage of both deciduous and evergreen shrubs increased in the combined warming and litter addition treatment, but no differences were found in 2012 (Table2). Of the individual shrub species, *A. polifolia* increased under warming and *R. lapponicum* in the combined *W* + *L* treatment in both years (Table S5). *B. nana* coverage increased by warming and litter addition separately (Table S5). The coverage of forbs was increased by the warming treatment, and the differences found between warming and other treatments were statistically significant in 2012 (Table2). The increase was due to changes in *A. alpinus* coverage (Table S5).

The coverage of lichens showed a drastic decrease in warming plus litter addition-treated plots compared to other plots (Table2). Litter addition alone also decreased the lichen coverage in 2010. The moss coverage was significantly higher in the combined treatment compared to other treatments in 2012.

### Covariance between vegetation and BVOC emissions

**Figure 5:**
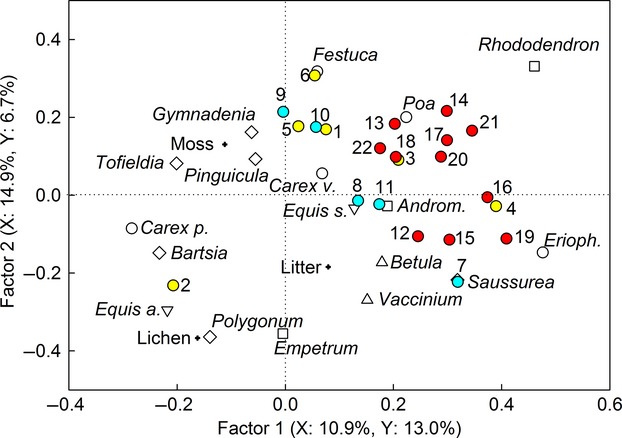
Partial least squares (PLS) regression on vegetation coverage and emissions of individual BVOCs. Graminoids (open circles): *Carex parallela*, *Carex vaginata*, *Eriophorum vaginatum*, *Festuca ovina*, *Poa alpigena*; forbs (diamonds): *Bartsia alpina*, *Pinguicula vulgaris*, *Polygonum viviparum*, *Saussurea alpina*, *Tofieldia pusilla*, *Gymnadenia conopsea*; vascular cryptogams (downward triangles): *Equisetum arvense*, *Equisetum scirpoides*; deciduous shrubs (upward triangles): *Betula nana*, *Vaccinium uliginosum*; evergreen shrubs (squares): *Andromeda polifolia*, *Empetrum hermaphroditum*, *Rhododendron lapponicum*; and lichens, mosses, litter (crosses). Other VOCs (1-6, yellow symbols): 2-methylfuran 1, 2-propenoic acid, 2-methyl-, methyl ester 2, benzene 3, cyclopentane 4, nonanal 5, toluene 6; monoterpenes (7–10, blue symbols): cymene 7, *α*-pinene 8, camphene 9, limonene 10, eucalyptol 11; sesquiterpenes (12–22, red symbols): aromadendrene 12, *α*-selinene 13, *β*-selinene 14, copaene 15, *δ*-cadinene 16, eudesma-3,7(11)-diene 17, germacrene 18, *γ*-muurolene 19, *γ*-selinene 20, *α*-caryophyllene 21, valencene 22. The explained variances of the independent (X, plant species) and dependent (Y, BVOCs) data are shown in parentheses. Data are from both 2010 and 2012, from the BVOC measurement closest to the vegetation analysis.

The PLS analysis mainly described the relationships between plant species abundances and BVOC emissions. The strongest correlation was found between the abundance of *R. lapponicum* and the emissions of SQTs, especially *α*-caryophyllene, *β*-selinene and eudesma-3,7(11)-diene (Fig.5). Negative correlation was found between the abundance of *R. lapponicum* and that of *E. arvense*, *Carex parallela*, lichens and the forbs *Polygonum viviparum* and *Bartsia alpina*. This group of species characterized plots with low SQT and high 2-methyl-2-propenoic acid methyl ester emissions relative to other plots (Fig.5).

The abundance of *E. hermaphroditum* correlated negatively with that of the graminoid *F. ovina*, which was present in plots with high relative emissions of the other VOCs toluene, nonanal and 2-methylfuran, and the MTs camphene and limonene (Fig.5). The plots with abundance of *B. nana* were characterized with the emissions of the MT cymene and several SQTs, for example *γ*-muurolene and copaene. These plots had a low relative abundance of mosses and the forb *T. pusilla*.

## Discussion

Our results show that the effects of experimental warming on BVOC emissions from a subarctic heath remain strong still after 13 years of treatment. While effects of warming were similar or larger than in earlier measurements, the litter addition treatment, which had not caused any clear effects during the first 7 years of the experiment (Tiiva *et al*., 2008; Faubert *et al*., 2010), had now translated into significant stimulatory effects on the emission of isoprene and other VOCs. In addition, the litter addition treatment enhanced the effects of warming on SQTs and other VOCs. The responses suggest that climate change in the subarctic gradually leads to vegetation changes that have implications for the amount and composition of the volatile emissions released.

We showed here that more than a decade of annual litter additions increased the emissions of isoprene from the studied subarctic heath, although there was large variation across measurements. In the previous measurements made after 6–7 treatment years (Tiiva *et al*., 2008; Faubert *et al*., 2010), only minor or no effects of litter were found, so we suggest that the response observed now is partly a result of changing plant species composition. The litter addition treatment has especially increased the coverage of the dwarf birch *B. nana*, the bog rosemary *A. polifolia* and the common horsetail *Equisetum arvense,* and decreased the coverage of the sedge *C. vaginata* as well as lichens. The growth of *B. nana*, *A. polifolia* and *E. arvense* may have been enhanced by litter addition that increased the availability of phosphorus and nitrogen concentrations for plants (Rinnan *et al*., 2007, 2008; Sorensen & Michelsen, 2011). *B. nana* has been shown to be a source of a variety of BVOCs, including many MTs and SQTs, but to lack isoprene emission (Rinnan *et al*., 2011). The presence of *A. polifolia* in boreal peatland microcosms has been suggested to be related to emissions of aromatic, carbonyl and terpenoid compounds (Rinnan *et al*., 2005). Thus, this increased coverage of *A. polifolia* and *B. nana* could partly explain the increased SQT emissions in 2012, but is most likely not related to the increased isoprene emission.

An alternative explanation to the increased emission of isoprene and other volatiles under litter addition is that the increased soil nutrient availability (Rinnan *et al*., 2007, 2008; Sorensen & Michelsen, 2011) may have stimulated the microbial production of BVOCs and/or abiotic release of volatiles from litter decomposition (Leff & Fierer, 2008; Ramirez *et al*., 2009).

Warming increased terpenoid emissions, as shown earlier by Faubert *et al*. (2010). They suggested that the emission increase was a direct effect of warming resulting from increased volatility of the stored compounds and stimulated *de novo* synthesis (Sharkey & Yeh, 2001; Loreto & Schnitzler, 2010), because a measurement conducted without the warming open-top chambers showed no treatment effects (Faubert *et al*., 2010). In order to test whether the increased emissions observed in our measurements were due to a direct warming effect, we minimized the momentary effects of temperature (and for isoprene PAR) differences by standardizing the emissions with the algorithms of Guenther *et al*. (1993, 1995). The effects of warming were still statistically significant after standardizing the emissions to the constant temperature and PAR, which could suggest that another factor than the direct effect of temperature or PAR would also affect the emissions.

There are many potential indirect effects of the long-term warming treatment. Our vegetation analysis showed that the coverage of forbs increased, and an earlier analysis of the normalized differential vegetation index (NDVI) has suggested increased biomass in the warming treatment (Rinnan *et al*., 2008). Of the individual species, the warming treatment favored *B. nana*, *A. polifolia*, *C. vaginata and A. alpinus* and in contrary decreased the coverage of *E. arvense*. According to our PLS analysis, the increased coverage of *B. nana* was found to correlate with emissions of the MT cymene and SQTs *γ*-muurolene and copaene. Hence, future warming might increase emissions of these compounds if *B. nana* coverage increases. However, this increase in taller *B. nana* shrubs is probably unfavorable to many prostrate species. In our study, the increase in *B. nana* was already found to correlate negatively with mosses and certain forb species, such as *T. pusilla*. In addition to vegetation composition changes, warming might drive changes in physiology or genetic traits of plants (Soudzilovskaia *et al*., 2013) and thus affect the BVOC compositions in future.

Combining warming with litter addition both caused the most drastic changes in vegetation composition and the highest increases in BVOC emissions. The large effects on the volatile emissions are likely due to vegetation responses to the alleviated temperature and nutrient limitation. Warming has been reported to increase carbon turnover in subarctic soils (Rinnan *et al*., 2007), and the more efficient decomposition processes with additional litter have increased the availability of phosphorus (Rinnan *et al*., 2007, 2008). Availability of nitrogen, one of the most important biomass-production-limiting factors in the subarctic, has also increased as both litter addition and warming considerably increase biological nitrogen fixation (Sorensen & Michelsen, 2011).

In these warming plus litter addition plots, the coverage of deciduous and evergreen shrubs and especially that of the Lapland rosebay *R. lapponicum* increased. This is in agreement with the results of Zamin *et al*. (2014), who observed a strong and consistent biomass increase in *Rhododendron subarcticum* in response to greenhouse warming of a mesic tundra in the Canadian low Arctic, and indicates that the genus *Rhododendron* is likely to increase in abundance in the changing Arctic. As *Rhododendron* was clearly related to SQT emissions according to our PLS analysis, and a related species which is also present in the Arctic, *Rhododendron tomentosum*, emits high concentrations of monoterpenes and sesquiterpenes, especially when the shoots are young (Butkiene *et al*., 2008), an increase in the genus *Rhododendron* would most likely lead to increased SQT emissions.

Combined warming and litter addition caused decrease in the lichen population. This decrease in lichen coverage was probably caused by increased shading from the increased density of the taller-growing vascular plants (Campioli *et al*., 2012) and was in agreement with previous studies (Graglia *et al*., 2001; Elmendorf *et al*., 2012b).

In 2012, a strong herbivory pressure caused by an outburst of *Epirrita autumnata*, autumnal moth, might partly explain the higher SQT emissions than in 2010. In addition to a range of products of the lipoxygenase pathway, herbivory damage of vegetation is known to induce sesquiterpenes emissions (Kessler & Baldwin, 2002; Holopainen & Gershenzon, 2010). The outbreak caused defoliation of a *B. pubescens* forest surrounding the heath, but did not occur in our experimental plots. Nevertheless, in previous studies it has been shown that BVOCs emitted from nearby plants, when they are under herbivory pressure, can act as signal compounds inducing the production of defensive BVOCs also in undamaged plants (Blande *et al*., 2014; Heil, 2014).

To conclude, we suggest that climate warming and increased leaf litter have both direct and indirect effects (through vegetation changes) on the BVOC emissions in the decadal time perspective. The 2-fold increase in MT and 5-fold increase in SQT emissions under the moderate open-top chamber warming were more than the doubling of the emission rates after 7 years (Faubert *et al*., 2010). This suggests that the changes in vegetation composition that have gradually taken place, especially with both warming and litter addition, become increasingly important drivers of the future BVOC emissions from the subarctic. The increasing amount of litter resulting from the changed vegetation appears to fertilize the soil alleviating nutrient limitation (especially when litter decomposition is accelerated by warming) of the plants and allowing for higher BVOC emissions from the higher plant biomass. We suggest that the changes in the subarctic vegetation composition induced by climate warming will be the major factor indirectly affecting the BVOC emission potentials and composition. We estimate that the relative contribution of the subarctic BVOC emissions to the global emissions will increase in the future. This may be especially important for the negative feedback effects via particle formation, as these effects are likely to be strongest in remote northern areas with low anthropogenic VOC emissions (Paasonen *et al*., 2013).

## Supporting Information

**Figure S1.** BVOC sampling. (a) A schematic drawing of the used equipment and (b) a photo of the enclosure chamber.

**Table S1.** Chamber temperature and photosynthetically active radiation (PAR; *μ*mol m^−2 ^s^−1^) during BVOC measurements (mean ± SE, *n *=* *6) in control, litter addition, warming and combined warming and litter addition (*W* + *L*) treatments.

**Table S2.** Emission rates of individual BVOCs from a subarctic tundra heath on 26 July, 2010 and 16 July, 2012.

**Table S3** Emission potentials for isoprene, monoterpenes and sesquiterpenes.

**Table S4.** anova table showing the treatment effects of litter addition (*L*), warming (*W*), and *W* × *L* interactions on the emission potentials of isoprene, monoterpenes and sesquiterpenes for each measurement date in 2010 and 2012.

**Table S5.** Vegetation coverage of vascular plant species in control (*C*), litter addition (*L*), warming (*W*) and warming + litter addition (*W* + *L*) treatments in August 2010 and 2012.
