# Supplementary material for: Climate change-induced vegetation change as a driver of increased subarctic biogenic volatile organic compound emissions
Source: Glob Chang Biol. 2015 May 21;21(9):3478–88. doi: 10.1111/gcb.12953 (PMC4676918; doi:10.1111/gcb.12953)
Supplement: Supplementary file 1 [file gcb0021-3478-sd1.docx]

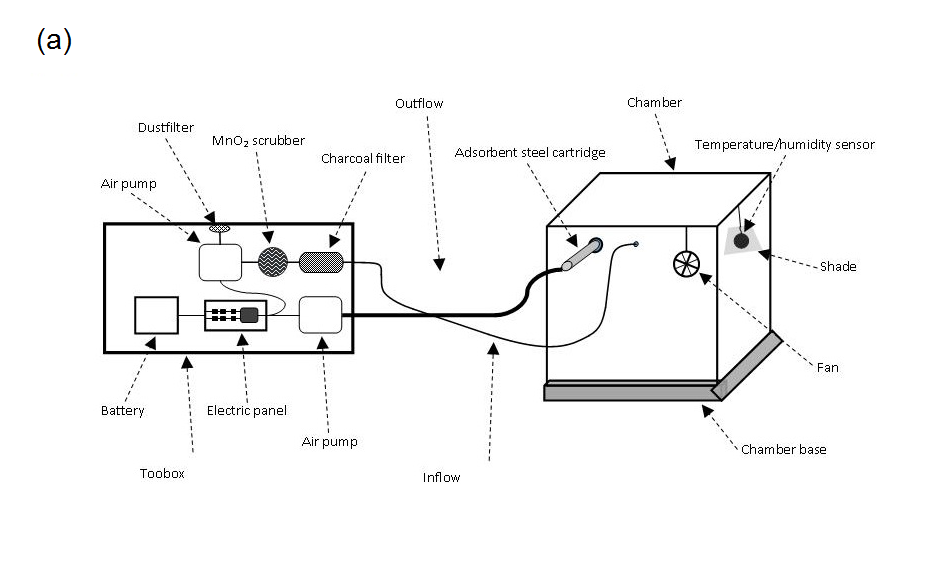


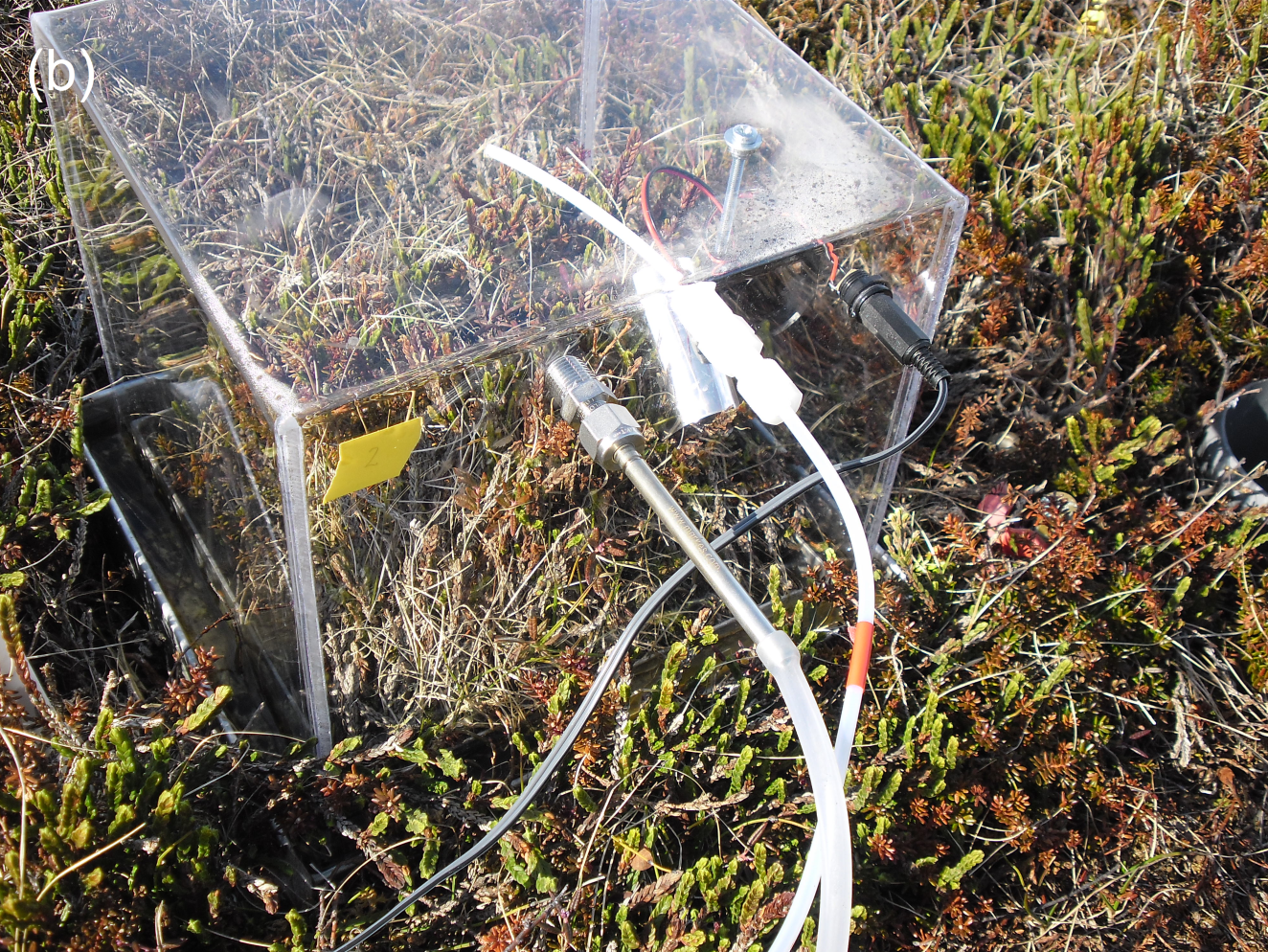


**Figure S1.** BVOC sampling. (a) A schematic drawing of the used equipment and (b) a photo of the enclosure chamber.
