# Supplementary material for: Climate change-induced vegetation change as a driver of increased subarctic biogenic volatile organic compound emissions
Source: Glob Chang Biol. 2015 May 21;21(9):3478–88. doi: 10.1111/gcb.12953 (PMC4676918; doi:10.1111/gcb.12953)
Supplement: Supplementary file 2 [file gcb0021-3478-sd2.docx]

**Table S1**. Chamber temperature and photosynthetically active radiation (PAR; µmol m^-2^s^-1^) during BVOC measurements (mean ± *SE*, *n* = 6) in control, litter addition, warming and combined warming and litter addition (W + L) treatments.

|  | |  | Chamber temperature (˚C) | | | | | | | | PAR (µmol m^-2^ s^-1^) | | | | | | | |
| --- | --- | --- | --- | --- | --- | --- | --- | --- | --- | --- | --- | --- | --- | --- | --- | --- | --- | --- |
|  | |  | Control | | Litter | | Warming | | W + L | | Control | | Litter | | Warming | | W + L | |
| 2010 | | |  |  |  |  |  |  |  |  |  |  |  |  |  |  |  |  |
|  | Jul 1 | | 21.3 ± 1.7 | | 21.9 ± 2.3 | | 19.6 ± 2.2 | | 22.3 ± 2.1 | | 992 ± 229 | | 822 ± 168 | | 520 ± 169 | | 730 ± 187 | |
|  | Jul 15 | | 21.5 ± 1.7 | | 25.8 ± 0.9 | | 24.2 ± 1.8 | | 28.6 ± 0.7 | | 987 ± 130 | | 1455 ± 43 | | 834 ± 118 | | 1170 ± 79 | |
|  | Jul 21 | | 17.8 ± 1.0 | | 17.5 ± 1.0 | | 19.3 ± 0.9 | | 19.9 ± 0.9 | | 613 ± 47 | | 635 ± 72 | | 495 ± 46 | | 489 ± 40 | |
|  | Jul 26 | | 27.4 ± 1.3 | | 24.1 ± 1.3 | | 27.8 ± 1.1 | | 25.9 ± 1.2 | | 1027 ± 112 | | 1057 ± 122 | | 840 ± 102 | | 729 ± 107 | |
|  | Aug 3 | | 17.8 ± 1.5 | | 19.2 ± 1.0 | | 19.9 ± 0.7 | | 20.9 ± 1.7 | | 592 ± 76 | | 648 ± 87 | | 381 ± 62 | | 475 ± 62 | |
|  | Aug 11 | | 20.2 ± 1.0 | | 21.6 ± 1.7 | | 22.2 ± 1.6 | | 21.6 ± 0.9 | | 674 ± 129 | | 798 ± 141 | | 366 ± 89 | | 387 ± 36 | |
|  | Aug 27 | | 12.2 ± 0.5 | | 11.5 ± 0.6 | | 12.9 ± 0.8 | | 12.0 ± 0.4 | | 295 ± 57 | | 281 ± 37 | | 219 ± 28 | | 235 ± 25 | |
|  | Sept 6 | | 13.7 ± 1.1 | | 13.3 ± 0.8 | | 14.4 ± 1.2 | | 14.2 ± 0.9 | | 348 ± 77 | | 347 ± 69 | | 295 ± 64 | | 310 ± 53 | |
| 2012 | | |  |  |  |  |  |  |  |  |  |  |  |  |  |  |  |  |
|  | Jun 14 | | 24.5 ± 1.0 | | 24.7 ± 1.2 | | 26.8 ± 0.9 | | 27.6 ± 1.0 | | 1190 ± 107 | | 1206 ± 125 | | 1057 ± 95 | | 1365 ± 149 | |
|  | Jun 28 | | 17.1 ± 2.6 | | 15.6 ± 2.4 | | 18.7 ± 3.2 | | 16.5 ± 2.1 | | 706 ± 269 | | 604 ± 244 | | 281 ± 62 | | 271 ± 55 | |
|  | Jul 16 | | 23.3 ± 1.3 | | 23.5 ± 1.9 | | 25.3 ± 2.1 | | 21.1 ± 1.3 | | 531 ± 124 | | 529 ± 127 | | 310 ± 94 | | 237 ± 28 | |
|  | Aug 20 | | 34.3 ± 1.1 | | 34.4 ± 1.8 | | 31.5 ± 0.6 | | 28.3 ± 1.5 | | 517 ± 31 | | 502 ± 43 | | 357 ± 40 | | 397 ± 70 | |
