# Supplementary material for: Climate change-induced vegetation change as a driver of increased subarctic biogenic volatile organic compound emissions
Source: Glob Chang Biol. 2015 May 21;21(9):3478–88. doi: 10.1111/gcb.12953 (PMC4676918; doi:10.1111/gcb.12953)
Supplement: Supplementary file 3 [file gcb0021-3478-sd3.docx]

**Table S2.** Actual emission rates of individual BVOCs from a subarctic tundra heath on 26 July, 2010 and 16 July, 2012 (µg m^-2^ h^-1^, mean ± *SE, n* = 6). Treatments are control (C), litter addition (L), warming (W) and combined warming and litter addition (W + L).

|  |  | C | L | W | W + L |
| --- | --- | --- | --- | --- | --- |
| 26 July 2010 | |  |  |  |  |
|  | α-pinene | 0 | 0 | 0.04 ± 0.00 | 0.01 |
|  | β-pinene | 0 | 0 | 0.01 ± 0.01 | 0.02 ± 0.02 |
|  | limonene | 0 | 0 | 0.04 ± 0.02 | 0.03 ± 0.03 |
|  | eucalyptol | 0.59 ± 0.43 | 0.68 ± 0.48 | 1.65 ± 0.78 | 1.75 ± 0.56 |
|  | cymene | 0 | 0 | 0.09 ± 0.05 | 0.08 ± 0.04 |
|  | β-cubebene | 0 | 0 | 0 | 0 |
|  | copaene | 0 | 0 | 0 | 0.12 ± 0.09 |
|  | aromadendrene | 0 | 0 | 0.03 ± 0.03 | 0.07 ± 0.04 |
|  | germacrene | 0 | 0.12 ± 0.12 | 0 | 0.10 ± 0.10 |
|  | 1.5.9.9-tetramethyl-1.4.7-cycloundecatriene | 0 | 0 | 0.05 ± 0.05 | 0.32 ± 0.23 |
|  | α-caryophyllene | 0 | 0.02 ± 0.02 | 0.22 ± 0.11 | 0.06 ± 0.05 |
|  | α-selinene | 0.04 ± 0.04 | 0.06 ± 0.06 | 0.60 ± 0.38 | 0.38 ± 0.29 |
|  | β-selinene | 0.42 ± 0.22 | 0.30 ± 0.25 | 1.13 ± 0.75 | 2.63 ± 1.52 |
|  | γ-selinene | 0.18 ± 0.18 | 0.21 ± 0.17 | 0.06 ± 0.06 | 0.28 ± 0.19 |
|  | γ-cadinene | 0 | 0 | 0 | 0 |
|  | δ-cadinene | 0.02 ± 0.02 | 0.02 ± 0.02 | 0.01 ± 0.01 | 0.02 ± 0.02 |
|  | eudesma-3.9.11-diene | 0.09 ± 0.09 | 0 | 0.11 ± 0.47 | 0.47 ± 0.22 |
|  | 2-methylfuran | 0 | 0 | 0.07 ± 0.03 | 0.01 ± 0.01 |
|  | benzene | 0 | 0 | 0 | 0 |
|  | xylene | 0 | 0 | 0 | 0 |
|  | 2-propenoicacid,2-methylmethylester | 0.09 ± 0.04 | 0 | 0.13 ± 0.05 | 0.09 ± 0.06 |
| 16 July 2012 | | C | L | W | W + L |
|  | isoprene | 16.46 ± 4.48 | 12.37 ± 5.55 | 6.41 ± 5.44 | 37.13 ± 18.43 |
|  | camphene | 0.03 ± 0.02 | 0 | 0.05 ± 0.03 | 0.21 ± 0.12 |
|  | 3-thujene | 0.47 ± 0.47 | 1.01 ± 1.01 | 0.08 ± 0.08 | 0 |
|  | α-pinene | 0.16 ± 0.16 | 0.11 ± 0.07 | 0.64 ± 0.39 | 0 |
|  | γ-terpinene | 0.03 ± 0.03 | 0 | 0.33 ± 0.33 | 0.14 ± 0.14 |
|  | limonene | 0.05 ± 0.05 | 0.06 ± 0.06 | 0.26 ± 0.15 | 0.07 ± 0.03 |
|  | eucalyptol | 0.47 ± 0.17 | 0.66 ± 0.29 | 1.47 ± 0.65 | 0.83 ± 0.28 |
|  | cymene | 0.19 ± 0.13 | 0.04 ± 0.04 | 0.40 ± 0.27 | 0.10 ± 0.10 |
|  | α-cubebene | 0.36 ± 0.19 | 0.10 ± 0.07 | 0.25 ± 0.13 | 0.35 ± 0.18 |
|  | copaene | 0.27 ± 0.27 | 0.41 ± 0.41 | 0.16 ± 0.16 | 0.06 ± 0.06 |
|  | γ-elemene | 0 | 0 | 0 | 0 |
|  | α-caryophyllene | 0.21 ± 0.13 | 0.39 ± 0.39 | 1.56 ± 1.17 | 1.45 ± 0.72 |
|  | aromadendrene | 0 | 0 | 0.14 ± 0.10 | 0 |
|  | γ-muurolene | 0.01 ± 0.01 | 0.04 ± 0.04 | 0.14 ± 0.11 | 0.02 ± 0.02 |
|  | β-cubebene | 0.06 ± 0.04 | 0 | 0.05 ± 0.03 | 0 |
|  | germacrene | 0.04 ± 0.04 | 0.30 ± 0.30 | 0.09 ± 0.05 | 0.10 ± 0.07 |
|  | α-selinene | 0.53 ± 0.30 | 0 | 1.50 ± 1.36 | 3.13 ± 1.42 |
|  | β-selinene | 0.70 ± 0.50 | 1.05 ± 0.91 | 3.58 ± 3.06 | 5.79 ± 2.73 |
|  | γ-selinene | 0 | 0.48 ± 0.41 | 0.19 ± 0.19 | 0.23 ± 0.17 |
|  | δ-cadinene | 0.04 ± 0.02 | 0.09 ± 0.09 | 0.09 ± 0.07 | 0.16 ± 0.07 |
|  | β-panasinsene | 0 | 0.09 ± 0.09 | 0 | 0.30 ± 0.30 |
|  | valencene | 0.22 ± 0.16 | 0 | 0.29 ± 0.29 | 0.09 ± 0.09 |
|  | eudesma-3.7.11-diene | 0.10 ± 0.10 | 0.11 ± 0.11 | 0.41 ± 0.35 | 0.75 ± 0.42 |
|  | 2-methylfuran | 0.13 ± 0.13 | 0.12 ± 0.12 | 0.36 ± 0.30 | 0.62 ± 0.36 |
|  | cyclopentane | 0 | 0.36 ± 0.23 | 0.34 ± 0.26 | 0 |
|  | nonanal | 0.58 ± 0.56 | 0.01 ± 0.01 | 0.26 ± 0.26 | 0 |
|  | p-xylene | 0 | 0 | 0.13 ± 0.13 | 0 |
|  | m-xylene | 0 | 0 | 0.13 ± 0.13 | 0.10 ± 0.10 |
|  | benzene | 0.36 ± 0.23 | 1.28 ± 0.81 | 1.06 ± 0.59 | 0.16 ± 0.16 |
|  | toluene | 1.55 ± 0.54 | 1.48 ± 0.58 | 1.36 ± 0.50 | 1.18 ± 0.42 |
|  | octene | 0 | 0 | 0.13 ± 0.13 | 0.07 ± 0.07 |
