# Supplementary material for: Climate change-induced vegetation change as a driver of increased subarctic biogenic volatile organic compound emissions
Source: Glob Chang Biol. 2015 May 21;21(9):3478–88. doi: 10.1111/gcb.12953 (PMC4676918; doi:10.1111/gcb.12953)
Supplement: Supplementary file 4 [file gcb0021-3478-sd4.docx]

**Table S3**. Standardized emission potentials (μg m^-2^ h^-1^, mean ± *SE, n* = 6) for isoprene, monoterpenes and sesquiterpenes. The data are emissions standardized to the temperature of 30°C and photosynthetically active radiation of 1000 μmol m^-2^s^-1^.

|  |  | Control | Litter | Warming | Warming + Litter |
| --- | --- | --- | --- | --- | --- |
| 2010 |  |  |  |  |  |
|  | *Monoterpenes* |  |  |  |  |
|  | July 1 | 0.2 ± 0.2 | <0.01 | 0.9 ± 0.7 | 0.2 ± 0.1 |
|  | July 15 | 2.0 ± 0.7 | 2.4 ± 1.4 | 3.7 ± 1.0 | 3.7 ± 1.2 |
|  | July 21 | 0.4 ± 0.4 | 0.6 ± 0.3 | 0.7 ± 0.6 | 0.4 ± 0.4 |
|  | July 26 | 0.8 ± 0.6 | 1.0 ± 0.6 | 1.9 ± 0.7 | 2.4 ± 0.7 |
|  | August 3 | <0.01 | 0.7 ±0.7 | 0.5 ± 0.5 | 0.5 ± 0.3 |
|  | August 11 | 1.1 ± 0.7 | 1.2 ± 0.9 | 2.1 ± 1.0 | 0.5 ± 0.3 |
|  | August 27 | <0.01 | <0.01 | <0.01 | <0.01 |
|  | September 6 | <0.01 | <0.01 | <0.01 | <0.01 |
|  | *Sesquiterpenes* |  |  |  |  |
|  | July 1 | 14.9 ± 12.9 | 0.3 ± 0.3 | 4.3 ± 1.3 | 18.2 ± 12.9 |
|  | July 15 | 12.3 ± 6.3 | 2.7 ± 1.9 | 4.8 ± 1.8 | 11.4 ± 5.1 |
|  | July 21 | 6.8 ± 3.4 | 3.3 ± 2.4 | 4.7 ± 2.8 | 53.8 ± 19.9 |
|  | July 26 | 1.0 ± 0.4 | 4.4 ± 4.0 | 4.7 ± 2.9 | 9.3 ± 3.0 |
|  | August 3 | 8.8 ± 8.3 | 5.5 ± 5.0 | 4.4 ± 2.5 | 17.6 ± 11.3 |
|  | August 11 | 1.7 ± 0.8 | 2.2 ± 2.1 | 6.0 ± 4.3 | 5.7 ± 4.7 |
|  | August 27 | <0.01 | 0.9 ± 0.9 | <0.01 | 0.5 ± 0.5 |
|  | September 6 | <0.01 | <0.01 | <0.01 | <0.01 |
| 2012 |  |  |  |  |  |
|  | *Isoprene* |  |  |  |  |
|  | June 14 | 15.2 ± 6.7 | 56.9 ± 34.0 | 143.0 ± 41.1 | 38.7 ± 17.5 |
|  | June 28 | 21.1 ± 14.0 | 84.7 ± 30.8 | 280.0 ± 165.9 | 93.3 ± 47.4 |
|  | July 16 | 47.0 ± 11.1 | 30.4 ± 15.9 | 63.2 ± 59.0 | 241.6 ± 142.9 |
|  | August 20 | 29.5 ± 18.6 | 333.8 ± 198.6 | 40.8 ± 25.6 | 237.5 ± 147.1 |
|  | *Monoterpenes* |  |  |  |  |
|  | June 14 | 0.1 ± 0.1 | 0.1 ± 0.1 | 2.7 ± 1.6 | 1.6 ± 0.4 |
|  | June 28 | 0.5 ± 0.2 | 0.4 ± 0.3 | 1.4 ± 0.6 | 1.6 ± 0.7 |
|  | July 16 | 2.3 ± 1.2 | 3.0 ± 1.8 | 4.3 ± 2.3 | 2.7 ± 0.9 |
|  | August 20 | 0.6 ± 0.1 | 1.9 ± 0.7 | 3.1 ± 0.8 | 4.2 ± 2.6 |
|  | *Sesquiterpenes* |  |  |  |  |
|  | June 14 | 5.0 ± 1.9 | 14.6 ± 8.8 | 54.2 ± 24.2 | 26.4 ± 11.7 |
|  | June 28 | 1.1 ± 0.7 | <0.01 | 57.2 ± 53.0 | 190.9 ± 130.6 |
|  | July 16 | 11.6 ± 5.2 | 8.3 ± 4.8 | 18.2 ± 11.4 | 51.4 ± 23.8 |
|  | August 20 | 0.5 ± 0.2 | 3.5 ± 2.9 | 13.6 ± 11.1 | 89.0 ± 37.3 |
