# Supplementary material for: Climate change-induced vegetation change as a driver of increased subarctic biogenic volatile organic compound emissions
Source: Glob Chang Biol. 2015 May 21;21(9):3478–88. doi: 10.1111/gcb.12953 (PMC4676918; doi:10.1111/gcb.12953)
Supplement: Supplementary file 5 [file gcb0021-3478-sd5.docx]

**Table S4.** ANOVA table showing the treatment effects of litter addition (L), warming (W) and W × L interactions on the emission potentials of isoprene, monoterpenes and sesquiterpenes for each measurement date in 2010 and 2012.

|  |  | L | W | W × L |
| --- | --- | --- | --- | --- |
| 2010 |  |  |  |  |
| *Monoterpenes* | |  |  |  |
|  | July 1 | 0.275 | 0.155 | 0.611 |
|  | July 15 | 0.356 | 0.092 + | 0.618 |
|  | July 21 | 0.818 | 0.519 | 0.546 |
|  | July 26 | 0.811 | 0.026 ↑ | 0.985 |
|  | August 3 | 0.306 | 0.407 | 0.812 |
|  | August 11 | 0.390 | 0.724 | 0.548 |
|  | August 27 | - | - | - |
|  | September 6 | - | - | - |
| *Sesquiterpenes* | |  |  |  |
|  | July 1 | 0.307 | 0.354 | 0.284 |
|  | July 15 | 0.081+ | 0.036 ↑ | 0.057 + |
|  | July 21 | 0.225 | 0.043 ↑ | 0.006 ↑ |
|  | July 26 | 0.457 | 0.060 + | 0.115 |
|  | August 3 | 0.406 | 0.080 + | 0.411 |
|  | August 11 | 0.537 | 0.182 | 0.719 |
|  | August 27 | 0.118 | 0.850 | 0.757 |
|  | September 6 | - | - | - |
| 2012 |  |  |  |  |
| *Isoprene* | |  |  |  |
|  | June 14 | 0.068 + | 0.140 | 0.161 |
|  | June 28 | 0.883 | 0.480 | 0.191 |
|  | July 16 | 0.513 | 0.504 | 0.017 ↑ |
|  | August 20 | 0.013 ↑ | 0.489 | 0.374 |
| *Monoterpenes* | |  |  |  |
|  | June 14 | 0.727 | <0.001↑ | 0.790 |
|  | June 28 | 0.620 | 0.023 ↑ | 0.528 |
|  | July 16 | 0.842 | 0.329 | 0.740 |
|  | August 20 | 0.956 | 0.235 | 0.743 |
| *Sesquiterpenes* | |  |  |  |
|  | June 14 | 0.700 | 0.041 ↑ | 0.709 |
|  | June 28 | 0.651 | 0.018 ↑ | 0.373 |
|  | July 16 | 0.648 | 0.145 | 0.386 |
|  | August 20 | 0.084 + | 0.026 ↑ | 0.650 |

↑, statistically significant increase in emissions (*p <* 0.050) or for interactions, higher emission in combined treatment than that expected from single treatments

+, nearly significant increases (0.05 < *p <* 0.10)

–, emission potentials are below detection limit.
