# Supplementary material for: Climate change-induced vegetation change as a driver of increased subarctic biogenic volatile organic compound emissions
Source: Glob Chang Biol. 2015 May 21;21(9):3478–88. doi: 10.1111/gcb.12953 (PMC4676918; doi:10.1111/gcb.12953)
Supplement: Supplementary file 6 [file gcb0021-3478-sd6.docx]

**Table S5.** Vegetation coverage (%, mean ± *SE*, *n* = 6) of vascular plant species in control (C), litter addition (L), warming (W) and warming + litter addition (W + L) treatments in August 2010 and 2012.

| **2010** | C | | L | W | W + L | *p-*value^1^ |
| --- | --- | --- | --- | --- | --- | --- |
| **Graminoids**  *Calamagrostis lapponica* | 0.0^a^ | 0.8 ± 0.8^b^ | | 0.0^a^ | 0.0^a^ | 0.011 |
| *Carex parallela* | 10.2 ± 3.3^a^ | 3.3 ± 1.6^b^ | | 5.7 ± 1.8^a^ | 3.3 ± 1.4^b^ | <0.001 |
| *Carex rupestris* | 1.0 ± 1.0 | 0.0 | | 0.0 | 0.0 |  |
| *Carex vaginata* | 19.0 ± 3.7^a^ | 11.5 ± 5.7^b^ | | 25.8 ± 7.3^a^ | 23.0 ± 4.0^a^ | <0.01 |
| *Eriophorum vaginatum* | 0.0 | 0.0 | | 0.0 | 0.0 |  |
| *Festuca ovina* | 0.0^a^ | 0.0^a^ | | 0.7 ± 0.7^b^ | 0.0^a^ | 0.004 |
| *Poa alpigena* | 0.0 | 0.0 | | 0.0 | 0.0 |  |
| **Forbs**  *Astragalus alpinus* | 0.7 ± 0.7^a^ | 0.0^b^ | | 17.5 ± 17.5^c^ | 0.0^b^ | 0.002 |
| *Astragalus frigidus* | 0.0^a^ | 0.0^a^ | | 0.0^a^ | 0.8 ± 0.8^b^ | <0.001 |
| *Bartsia alpina* | 0.3 ± 0.3 | 0.5 ± 0.5 | | 0.8 ± 0.8 | 0.3 ± 0.3 |  |
| *Cerastium alpinum* | 0.0 | 0.0 | | 0.0 | 0.0 |  |
| *Chamorchis alpina* | 0.0^a^ | 0.0^a^ | | 0.0^a^ | 0.2 ± 0.2^b^ | 0.005 |
| *Gymnadenia conopsea* | 0.0 | 0.0 | | 0.0 | 0.0 |  |
| *Leucorchis albida* | 0.0^a^ | 0.0^a^ | | 0.17 ± 0.17^b^ | 0.0^a^ | 0.015 |
| *Pedicularis lapponica* | 0.0 | 0.0 | | 0.0 | 0.0 |  |
| *Pinguicula vulgaris* | 0.0 | 0.0 | | 0.3 ± 0.2 | 0.7 ± 0.7 |  |
| *Polygonum viviparum* | 1.7 ± 0.6 | 1.5 ± 0.6 | | 0.5 ± 0.2 | 1.2 ± 0.5 |  |
| *Rubus chamaemorus* | 0.0 | 0.0 | | 0.0 | 0.0 |  |
| *Saussurea alpina* | 0.0 | 0.0 | | 0.0 | 0.0 |  |
| *Silena acaulis* | 0.0^a^ | 5.5 ± 5.5^b^ | | 0.0^a^ | 0.0^a^ | <0.001 |
| *Tofieldia pusilla* | 1.5 ± 0.7 | 1.8 ± 1.1 | | 2.2 ± 1.1 | 3.3 ± 1.7 |  |
| **Deciduous shrubs**  *Arctostaphylos alpinus* | 1.7 ± 0.6^a^ | 3.8 ± 3.1^b^ | | 1.3 ± 1.0^ab^ | 0.2 ± 0.2^b^ | 0.003 |
| *Betula nana* | 0.8 ± 0.5^a^ | 5.8 ± 4.3^a^ | | 10.0 ± 7.1^b^ | 0.7 ± 0.7^a^ | 0.011 |
| *Salix hastata* | 0.0^a^ | 0.0^a^ | | 0.2 ± 0.2^b^ | 2.5 ± 2.5^b^ | 0.023 |
| *Salix myrsinites* | 2.8 ± 2.8^a^ | 0.0^b^ | | 0.0^b^ | 0.0^b^ | 0.006 |
| *Salix reticulata* | 1.3 ± 1.0^a^ | 0.3 ± 0.2^b^ | | 0.3 ± 0.2^b^ | 0.2 ± 0.2^b^ | <0.001 |
| *Vaccinium uliginosum* | 12.8 ± 5.7^a^ | 10.3 ± 2.0^a^ | | 19.3 ± 6.0^b^ | 26.0 ± 6.6^b^ | <0.001 |
| **Evergreen shrubs**  *Andromeda polifolia* | 8.3 ± 1.7^a^ | 11.8 ± 2.7^b^ | | 20.5 ± 1.8^c^ | 16.7 ± 3.3^d^ | <0.001 |
| *Dryas octopetala* | 0.0 | 0.0 | | 0.0 | 0.0 |  |
| *Empetrum hermaphroditum* | 29.0 ± 9.1 | 29.8 ± 10.0 | | 22.0 ± 8.9 | 35.3 ± 18.3 |  |
| *Rhododendron lapponicum* | 4.5 ± 2.1^a^ | 5.8 ± 3.7^a^ | | 4.2 ± 1.2^a^ | 13.3 ± 5.7^b^ | 0.003 |
| *Saxifraga oppositifolia* | 0.0 | 0.0 | | 0.0 | 0.0 |  |
| *Vaccinium vitis-idaea* | 0.0 | 0.0 | | 0.0 | 0.0 |  |
| **Vascular cryptogams** |  |  | |  |  |  |
| *Equisetum arvense* | 3.3 ± 1.2^a^ | 2.3 ± 1.3^ab^ | | 1.0 ± 1.0^b^ | 4.8 ± 4.1^ab^ | 0.009 |
| *Equisetum scirpoides* | 0.3 ± 0.3^a^ | 2.3 ± 1.2^b^ | | 1.8 ± 1.1^b^ | 1.7 ± 0.8^b^ | <0.001 |
|  |  |  | |  |  |  |
| **2012** | C | L | | W | W + L | *p-*value^1^ |
| **Graminoids**  *Calamagrostis lapponica* | 0.0 | 0.0 | | 0.0 | 0.0 |  |
| *Carex parallela* | 1 ± 0.4 | 1 ± 0.5 | | 0.7 ± 0.5 | 1.2 ± 0.8 |  |
| *Carex rupestris* | 0.0 | 0.0 | | 0.0 | 0.0 |  |
| *Carex vaginata* | 16.0 ± 4.4^a^ | 6.7 ± 3.1^b^ | | 19.8 ± 5.6^c^ | 16.7 ± 2.1^ac^ | <0.001 |
| *Eriophorum vaginatum* | 0.8 ± 0.8^a^ | 0.7 ± 0.5^a^ | | 0.3 ± 0.3^a^ | 0.0^b^ | 0.045 |
| *Festuca ovina* | 6.8 ± 3.4 | 2.0 ± 1.1 | | 2.7 ± 1.7 | 1.2 ± 0.8 |  |
| *Poa alpigena* | 0.0 | 0.7 ± 0.7 | | 1.2 ± 1.2 | 0.3 ± 0.3 |  |
| **Forbs**  *Astragalus alpinus* | 1.5 ± 1.2^a^ | 0.0^b^ | | 8.2 ± 8.2^a^ | 0.0^b^ | 0.001 |
| *Astragalus frigidus* | 0.0 | 0.0 | | 0.0 | 0.0 |  |
| *Bartsia alpina* | 0.3 ± 0.3^a^ | 1.8 ± 1.2^a^ | | 1.7 ± 1.7^a^ | 0.0^b^ | 0.032 |
| *Cerastium alpinum* | 0.0^a^ | 0.0^a^ | | 0.0^a^ | 0.2 ± 0.2^b^ | 0.004 |
| *Chamorchis alpina* | 0.0 | 0.0 | | 0.0 | 0.0 |  |
| *Gymnadenia conopsea* | 0.3 ± 0.2 | 1.0 ± 1.0 | | 1.0 ± 0.7 | 0.2 ± 0.2 |  |
| *Leucorchis albida* | 0.0 | 0.0 | | 0.0 | 0.0 |  |
| *Pedicularis lapponica* | 0.0^a^ | 0.3 ± 0.3^b^ | | 0.0^a^ | 0.2 ± 0.2^b^ | 0.035 |
| *Pinguicula vulgaris* | 0.5 ± 0.3 | 0.5 ± 0.3 | | 0.3 ± 0.3 | 0.2 ± 0.2 |  |
| *Polygonum viviparum* | 0.2 ± 0.2 | 0.3 ± 0.3 | | 0.2 ± 0.2 | 0.7 ± 0.7 |  |
| *Rubus chamaemorus* | 0.0 | 0.0 | | 0.0 | 0.0 |  |
| *Saussurea alpina* | 1.0 ± 1.0 | 0.8 ± 0.5 | | 0.2 ± 0.2 | 0.5 ± 0.5 |  |
| *Silena acaulis* | 0.0 | 0.0 | | 0.0 | 0.0 |  |
| *Tofieldia pusilla* | 1.3 ± 0.4 | 4.0 ± 1.8 | | 3.0 ± 1.1 | 2.2 ± 0.8 |  |
| **Deciduous shrubs**  *Arctostaphylos alpinus* | 3.3 ± 2.0 | 3.5 ± 2.6 | | 2.0 ± 0.7 | 1.2 ± 1.0 |  |
| *Betula nana* | 1.0 ± 1.0^a^ | 4.5 ±3.0^b^ | | 12.8 ± 9.3^b^ | 1.7 ± 1.7^a^ | 0.008 |
| *Salix hastata* | 2.0 ± 2.0^a^ | 0.0^b^ | | 0.0^b^ | 0.5 ± 0.5^a^ | 0.029 |
| *Salix myrsinites* | 0.0^a^ | 0.0^a^ | | 0.0^a^ | 0.7 ± 0.7^b^ | 0.004 |
| *Salix reticulata* | 1.3 ± 0.8^a^ | 0.0^b^ | | 1.2 ± 0.8^a^ | 1.7 ± 1.7^a^ | 0.019 |
| *Vaccinium uliginosum* | 18.5 ± 3.2 | 13.8 ± 3.3 | | 16.7 ± 5.2 | 18.5 ± 6.2 |  |
| **Evergreen shrubs**  *Andromeda polifolia* | 14.3 ± 3.0^a^ | 19.7 ± 5.3^a^ | | 30.5 ± 3.1^b^ | 16.3 ± 2.9^a^ | <0.001 |
| *Dryas octopetala* | 0.0^a^ | 0.0^a^ | | 0.0^a^ | 0.3 ± 0.3^b^ | 0.004 |
| *Empetrum hermaphroditum* | 26.5 ± 8.8 | 31.5 ± 11.2 | | 20.5 ± 8.9 | 24.5 ± 11.1 |  |
| *Rhododendron lapponicum* | 6.2 ± 2.6^a^ | 7.5 ± 3.5^a^ | | 4.8 ± 1.6^a^ | 13.5 ± 4.0^b^ | 0.009 |
| *Saxifraga oppositifolia* | 0.0^a^ | 3.3 ± 3.3^b^ | | 0.3 ± 0.3^b^ | 0.0^a^ | 0.041 |
| *Vaccinium vitis-idaea* | 0.0^a^ | 0.0^a^ | | 0.0^a^ | 0.7 ± 0.4^b^ | <0.001 |
| **Vascular cryptogams** |  |  | |  |  |  |
| *Equisetum arvense* | 1.7 ± 1.1^a^ | 0.5 ± 0.5^a^ | | 0.0^b^ | 0.0^b^ | 0.001 |
| *Equisetum scirpoides* | 2.8 ± 1.6 | 6.2 ± 2.7 | | 6.3 ± 3.3 | 7.0 ± 3.3 |  |

^1^statistically significant treatment effects by Kruskall-Wallis test. Different letters within a plant species show significant differences between treatments (*p* < 0.05, Mann-Whitney test with Bonferroni correction).
